# Predictors of Language Dominance: An Integrated Analysis of First Language Attrition and Second Language Acquisition in Late Bilinguals

**DOI:** 10.3389/fpsyg.2018.01306

**Published:** 2018-08-20

**Authors:** Monika S. Schmid, Gülsen Yılmaz

**Affiliations:** ^1^Centre for Research on Language Development throughout the Lifespan (LaDeLi), Department of Language and Linguistics, University of Essex, Colchester, United Kingdom; ^2^Institut für Anglistik und Amerikanistik, Humboldt-Universität zu Berlin, Berlin, Germany

**Keywords:** bilingual development, language attrition, second language development, late bilinguals, language dominance, language balance, extralinguistic factors, non-linear statistical models

## Abstract

Late bilinguals who spend (part of) their adult lives in an environment where a language other than the one they learned in childhood is spoken typically experience a range of language development phenomena. Most obviously, they will acquire some level of receptive and/or productive knowledge of the new, or second, language (L2). How basic or advanced that level will be is determined by a range of environmental, experiential, attitudinal and individual factors. Secondly, they will most likely find the knowledge of their native language (L1) beginning to diverge from that of monolingual speakers in their country of origin, a process known as *language attrition*. In the course of this developmental process, some L2 skills may eventually match or even overtake the corresponding skill in the L1. This shift in the balance between L1 and L2 is the focus of investigations of *language dominance*. The present study explores language dominance in four migrant populations (Germans in the Netherlands and Canada, Turks and Moroccans in the Netherlands). Investigating both the development of formal/controlled skills and more automatic aspects of lexical access and fluency, we aim to attain an understanding of how extralinguistic factors contribute to the development of *both* languages. We argue that an integrated perspective can contribute more profound insights into the predictors of this complex process of bilingual development. In particular, our findings show that statistical models based on linear relationships fall short of capturing the full picture. We propose an alternative method of analysing data, namely discriminant function analysis, based on a categorisation of the populations, and demonstrate how this can enhance our understanding. Our findings suggest that different aspects of the bilingual experience contribute differently to language development, regardless of language combination and type of skill measured. Contrary to what previous research suggests, measures relating to the intensity of informal use of both the L1 and the L2 in daily life are important in determining whether someone is a good or a poor L1 maintainer, while high vs. low success in acquisition appears to be predominantly associated with personal factors such as educational level.

## Introduction

Language dominance is an extremely complex concept, encompassing a wide range of aspects and features (e.g., Gertken et al., 2014; Silva-Corvalan and Treffers-Daller, 2016). These features can roughly be divided into two sets: the first set consists of those aspects of language that usually constitute the outcome measures or dependent variables for linguistic investigations – that is, measurable phenomena that relate to the knowledge, use and processing of all of a bilingual’s languages at all linguistic levels – and fall under the broad concept of ‘proficiency.’ The second set comprises measures related to personal background variables such as age, education, or language aptitude; the context in which the languages were acquired; language experience and habits; and linguistic and cultural identification. These factors are usually the independent variables, they predict the extent to which the first set of variables is developed in any one individual speaker. Language dominance, therefore, “takes into account the two languages of a bilingual person, not just one, biographical variables and the language-external conditions under which the two languages are learned or used by bilinguals” (Montrul, 2016, p. 17).

**FIGURE 1:**
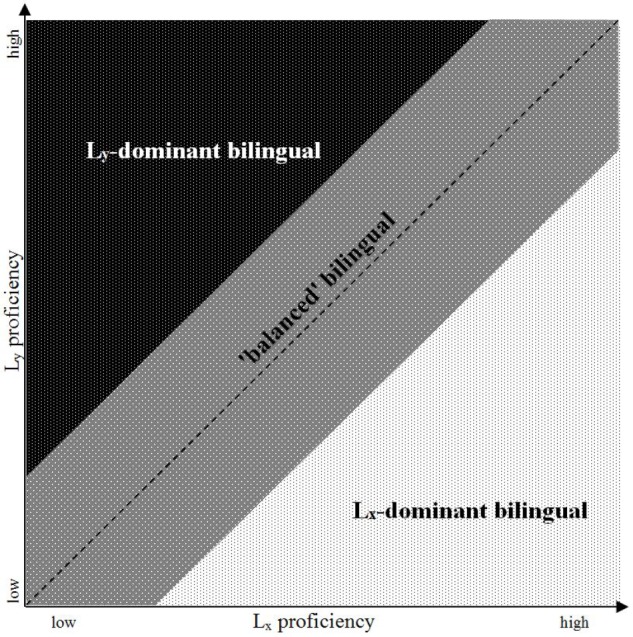
A schematic representation of language dominance in bilinguals.

In terms of the first factor set, which for the sake of simplicity we will refer to here as ‘proficiency measures,’ every bilingual speaker can therefore be situated somewhere in a two-dimensional space defined by an *x*-axis representing language *X* (*L*_x_) and a *y*-axis, representing language *Y* (*L*_y_) (see **Figure 1**)^1^. A speaker who is mapped close to the diagonal of this space (that is, at a similar level on both axes) is someone whose proficiency is more or less ‘balanced’ between the two languages, while one who is closer to one axis than the other is ‘dominant’ in the ‘stronger’ language, the one in which s/he has scored more highly. It should be noted that this visualisation is a simplified and idealised one: ‘proficiency’ cannot easily be reduced to a single measure (see section “Outcome Variables in Bilingual Development: Definitions and Measurements of ‘Proficiency”’ below), and the position of the same individual may therefore vary considerably depending on what skill or task is being measured (e.g., Bahrick et al., 1994; Kupisch and van de Weijer, 2016; Montrul, 2016).

The predictor variables are equally problematic in terms of definition and measurement, as this factor set covers a wide range of aspects of the bilingual experience and is therefore as varied as are the bilingual individuals themselves. Various models attempt to capture the multi-facetedness and multi-dimensionality of language exposure and use, for example the *Complementarity Principle* which assigns each domain of use – such as the home, politics, specific leisure activities etc. – one or several languages associated with it in a particular bilingual individual’s experience, reflecting the recognition that “[d]ifferent aspects of life require different languages” (Grosjean, 1997, p. 165; see also Grosjean, 2016). A ratio-based calculation (similar to the one used to establish handedness), based on absolute proficiency in both languages and capable of capturing language dominance as a gradient phenomenon, is described in Birdsong’s (2016) proposal of a *Dominance Index*.

The third dimension in the imaginary space defined by proficiency in *L*_x_ and *L*_y_ in **Figure 1** is *time*: development unfolds as the independent variables exert their influence on the dependent ones (e.g., when an increase in exposure to *L*_y_ leads to a higher level of proficiency in that language, potentially also affecting *L*_x_). Time, in this model, is the only dimension which is linear and unidirectional. Linguistic development is neither: it can shift toward the higher or the lower end of the spectrum in either language, encompassing both acquisition and loss. Such shifts can occur in bursts or slowly and gradually, they can reverse direction from growth to decline and back again and they can affect mainly one language, both languages equally, or both languages orthogonally (a growth in one language occurring alongside a decline in the other).

The formidable task for investigations of language dominance, then, is to provide explanatory models capable of mapping out how the predictor variables may interact and determine the intensity and direction of developmental changes for any given aspect of language proficiency over time. Crucially, we argue here that it is important to take into account *both* linguistic dimensions in order for such models to fully capture the phenomenon and not to reduce the analysis to the development of one language only, nor to collapse them into a one-dimensional function, e.g., by subtracting one from the other.

## Investigations of Development in Both Languages

To date, such integrated models of bilingual development have been strongly biased toward speakers for whom development in both languages goes hand in hand: simultaneous or early bilinguals for whom acquisition of *L*_y_ begins while *L*_x_ is still at an early stage of development. Findings indicate that the highly active phases of language development during childhood and adolescence allow great flexibility when it comes to shifts in language proficiency and dominance, with changes in external circumstances or exposure – such as the start of (nursery) school, a move between countries, or a return to the home country – often causing spurts of development and/or regression that may fundamentally change the overall multilingual balance within months (e.g., Flores, 2015; de Houwer and Bornstein, 2016).

In late bilingualism, on the other hand, the most intensive stages of development in each of an individual’s languages take place during different life phases, with the onset of second language acquisition (SLA) occurring after the development of the L1 has reached a relatively mature level and L1 development has hence slowed down considerably. That being the case, the majority of investigations of late bilingual development focus on the L2, the assumption being that a level of stability – some kind of ‘steady state’ – has been reached in the L1 which makes it uninteresting for research (e.g., Gregg, 2010). This notion has been challenged in the context of research on *first language attrition* (L1At) which argues that the addition of a new language will inevitably lead to changes in all of those aspects that we subsume here under the term *proficiency* in the language that is already established. A growing body of research provides evidence of such attrition in immersed as well as non-immersed bilinguals (e.g., Schmid and Köpke, 2017) and attempts to probe the relationship between predictor and outcome variables in this process (e.g., Schmid and Dusseldorp, 2010).

An immediate question in this respect is to what extent a gain with respect to any particular aspect of proficiency in one of a bilinguals’ languages may be associated with (or even cause) a loss in the other. When it comes to simultaneous or early bilingualism, popular understanding often has it that Language *X* will grow at the expense or to the detriment of Language *Y*. Research on bilingual development has long since demonstrated that there is no such straightforward or trivial relationship (e.g., Cummins, 1991; Bialystok, 2001). Bilingual children may develop both of their languages at the same pace, hitting the milestones assumed for typically developing monolinguals at roughly the same age in both, or development may be quite asymmetrical, favouring one language over the other at different stages. A developing bilingual child may occupy any position in the imaginary space mapped out in **Figure 1** above.

When it comes to L1At, findings on the relationship between proficiency in the speaker’s two languages are quite sketchy. In early research a relatively straightforward correlation was often assumed, according to which “[t]he greater the degree to which a speaker masters one system, the greater the extent to which one might expect it to affect another system,” so that “we expect L1 loss to be greatest in individuals who […] have mastered the L2 to a relatively high degree” (Major, 1992, p. 191). Opitz (2011) ascribes this hypothesised ‘trade-off’ in proficiency to the languages “competing for potentially insufficient resources required for maintaining the languages simultaneously at the desired high level” (p. 81). Other studies, however, have hypothesised that the development of proficiency in both L1 and L2 may be less straightforward, highly task-dependent, and modulated by other factors – for example, a high level of language aptitude may allow a particular speaker to acquire and maintain high proficiency levels in both languages (e.g., Cummins, 1991; Bylund et al., 2009; Cherciov, 2013).

There are few studies to date which investigate proficiency in both the L1 and the L2 of late immersed bilinguals. The findings that do attempt to make direct comparisons across languages and tasks (e.g., Dostert, 2009; Opitz, 2010, 2011, 2013; Cherciov, 2011) or link the amount of L1 attrition to the level of proficiency in the L2 (e.g., Baladzhaeva, 2013) seem to suggest that, similar to the early bilinguals discussed above, L1 attriters can fall anywhere on the spectrum: while in some cases (weak) correlations are observed between measures in both languages, a look at individual data suggests that this relationship is anything but deterministic. In other words, some participants attain high proficiency in the L2 but perform poorly in the L1, for others it is the other way around, while yet others are extremely good or quite poor in both their languages. This poses a problem for empirical investigations, since it implies that establishing language dominance based on difference scores (as is extremely common, e.g., the overview in Treffers-Daller, 2016) falls short of capturing the full picture. Such an approach ranks someone who does extremely well in both languages (a good maintainer and good learner) exactly the same as someone who performs very poorly across the board (a poor maintainer and poor learner) (see also Birdsong, 2016).

Treffers-Daller (2016, p. 261) takes this problem into account in her ‘typology of language dominance based on language proficiency’ which, instead of dividing the proficiency spectrum shown in **Figure 1** above into three sections (*L*_x_-dominant, *L*_y_-dominant and balanced) uses four quadrants: dominant bilinguals (either *L*_x_ < *L*_y_ or *L*_x_ > *L*_y_), low-achieving balanced bilinguals and high-achieving balanced bilinguals. Treffers-Daller (2016, p. 262) acknowledges, however, that this typology lacks explanatory value, since it “does not indicate how proficiency in these languages has developed”. She argues that future research agendas should therefore focus on the interaction between language use and ability in order to address this knowledge gap. A qualitative approach to doing so is suggested by Opitz (2016, in press) through scrutinising particularly good exemplars of the four developmental types. While such an approach is useful for gaining preliminary insights into the developmental processes and the factors which drive them, we propose that avenues should also be explored which allow for the quantitative/experimental exploration of large datasets in order to empirically verify such observations. The present study is a tentative attempt at one such approach.

## Problems of Measurement

### Predictor Variables in Bilingual Development

Where second language development is concerned, quality and quantity of *input* and *output* are among the most important predictors (e.g., Gass and Mackey, 2007) with a number of modulating variables linked to factors that are usually referred to as *individual differences*, such as motivation, aptitude and cognitive style (e.g., Dörnyei, 2005). The picture is much more obscure when it comes to the impact of predictor variables on L1At (e.g., Opitz, in press). A number of hypotheses have been advanced in this context, the most common one being that a lack of exposure to and use of the L1 will lead to its deterioration (e.g., Cook, 2003) – in other words, that the amount of L1At to be observed will correlate negatively with the degree of use of that language in daily life, and that these effects will increase with a longer period of residence in the host country. This hypothesis echoes the relationship between use and success observed in SLA. Secondly, and also in line with what has been found for SLA, it has often been predicted that attrition will be modulated by attitude and motivation, a more positive attitude toward the language itself and the speech community facilitating language maintenance (e.g., Cherciov, 2013). While both of these predictions may appear self-evident, empirical research to date has been strikingly unsuccessful in substantiating any such relationships (see Cherciov, 2011, 2013; Schmid, forthcoming for reviews) suggesting that, if there is an impact at all of exposure/use and attitude/motivation on L1At, it is much less pronounced and/or more complex than it is in SLA. In particular, while re-immersion in a monolingual context appears to help regain native-like levels on some aspects of proficiency that were shown to be attrited prior to the re-exposure (e.g., Chamorro et al., 2016; Genevska-Hanke, 2017) L1 use in daily life in the immigration setting has not been shown to be systematically related to performance (Schmid, forthcoming).

This difficulty of empirically establishing a connexion between predictor and outcome variables in the process of L1At has sometimes been ascribed to the assumption that the influence of neither individual predictors nor their interaction may produce an effect that is linear (e.g., de Bot et al., 1991; Schmid, 2011b) and that it may thus elude capture by means of traditional statistical techniques. A number of inevitable practical and methodological considerations further complicate matters: the multi-facetedness of the bilingual experience – particularly in the immersion setting typical for attrition studies – necessitates that the research design should include a large number of independent variables (see Schmid, 2011a). Many of these have to rely on self-assessments, usually elicited by means of Likert-scale type questions, and they are almost invariably non-normally distributed. For example, within the much-studied communities of the traditional ‘guest worker’ immigrants who arrived in Western European countries in the 1960s and 70s due to labour shortages, few speakers will report that they often use their native language for professional purposes, so this variable is likely to be skewed toward the L2 for such populations. In general it is, in our experience, quite rare for individual speakers to state that they use both languages equally in any one domain. This is a natural consequence of Grosjean’s Complementarity Principle (see above) – bilinguals use different languages to do different things – but it implies that most predictors will either be skewed toward one or the other language or show a bimodal distribution within communities that are less homogenous. While such problems of multifacetedness, non-linearity of interactions and non-normality of distributions have to be acknowledged as probably inevitable complicating considerations for language attrition research, we propose here that they can be dealt with by choosing the appropriate statistical procedures.

Beyond such methodological and practical considerations, however, we suggest that the lack of insight may be related to the fact that, to date, most of the empirical and quantitative work on L1At has limited itself to investigating the L1 (see Opitz, 2013), while studies of L2 development tend to only be interested in the L2, assuming the L1 to be an invariate baseline. The present study seeks to investigate to what extent our understanding can benefit from an approach that investigates language proficiency as a fully two-dimensional construct. This begs the question of what we understand by ‘language proficiency.’

### Outcome Variables in Bilingual Development: Definitions and Measurements of ‘Proficiency’

The problems and considerations listed above represent a formidable set of challenges for investigations of language dominance – they pale, however, in comparison with the difficulties involved in defining and measuring the elusive notion of ‘language proficiency.’ This is a catch-all term that has been used to describe radically different aspects of language skills and measurements, depending on the population under investigation and the theoretical framework within which a study is conducted (see Hulstijn, 2012 for an overview). For example, proficiency has been operationalised as mean length of utterance (MLU) in investigations of child language development (e.g., Yip and Matthews, 2006), as the ability to fill in the gaps in a cloze test in instructed second language learning (Tremblay, 2011), as the ability to pass as a native speaker in investigations of maturational limits to ultimate attainment (e.g., Bongaerts et al., 1997), as the ability to name objects on a computer screen quickly and accurately in investigations of language processing (e.g., Mägiste, 1992), as the ability to use the language with native-like levels of fluency (e.g., de Jong et al., 2015) or as the ability to recognise and process violations of particular grammatical features in studies of the development of underlying mental grammars and representations (e.g., Hopp, 2007) – to name but a few.

Studies of language dominance, which attempt to assess relative levels of proficiency across languages, first of all have to acknowledge that some of the skills or measures listed above lend themselves more readily to direct crosslinguistic comparisons of *L*_x_ and *L*_y_ development (for example, MLU or naming latencies are often used to establish levels of language dominance) while for others it is more difficult to see how crosslinguistic equivalence can be established (see Hulstijn, 2012; Montrul, 2016; Treffers-Daller, 2016 for discussion). In particular, specific questions of grammatical development (based, for example, on theoretical issues concerning parametrisation, interfaces etc.) are hard to address in such a framework, as by their very nature they will focus on features which are hard to acquire or maintain in only one of a bilingual’s languages, making meaningful comparisons across both dimensions hard. For investigations which aim to provide an integrated model of (global) proficiency in *L*_x_ and *L*_y_ among late bilinguals, methods which focus on tasks or measurements related to particular aspects of the structure of each language (such as grammaticality judgments or the production/perception of certain phonemes) are therefore problematic. Instead, the outcome variables should be selected to represent relatively general and holistic aspects of language proficiency, taking into account the extent to which these may vary crosslinguistically in both native and non-native populations.

Given that L1At populations command two languages which are learned under similar conditions (naturalistic learning through immersion in the linguistic community) but at different stages in life – that is, speakers who unambiguously have one native and one non-native language – a fruitful framework for the assessment of proficiency is the model proposed by Hulstijn (2011, 2015) which distinguishes Shared/Basic Language Cognition (BLC) and Extended/Higher Language Cognition (HLC). In this model, BLC refers to

(a) the largely implicit, unconscious knowledge in the domains of phonetics, prosody, phonology, morphology and syntax; (b) the largely explicit, conscious knowledge in the lexical domain (form-meaning mappings), *in combination with* (c) the automaticity with which these types of knowledge can be processed (Hulstijn, 2011, p. 230, his emphasis)

and is restricted to those lexical items and morphosyntactic structures in spoken language which all adult L1 speakers share (irrespective of their age, educational level, or level of literacy) and which they may encounter in all communicative situations. HLC, on the other hand, refers to more complex domains of use, encompassing less frequent items and structures as well as written language, and it is in this domain that native as well as non-native speakers vary considerably from each other. ‘Frequency,’ in this framework, is operationalised on the basis of the assumption that lexical items and grammatical structures follow a Zipfian distribution in naturally occurring language, where ‘highly frequent’ items (those belonging to BLC) are situated on the steep left side of the slope, while less frequent items are to be found on the flattening part of the curve to the right (Hulstijn, 2015:22ff.)

Among the fundamental assumptions of the model are

(a) Command of BLC should be homogenous within as well as across native populations, while these populations will be stratified when it comes to HLC.
(b) Non-native populations will exhibit considerable variability with respect to both; they are unlikely to fully acquire BLC in their L2 beyond the domains of vocabulary but may nonetheless reach high native levels of HLC
(c) Development of BLC in non-natives and HLC in both populations will be modulated by a combination of external and background factors (Hulstijn, 2015:52f.)

Hulstijn’s model thus assumes two types of speakers: the ‘native speaker,’ who will be at ceiling for all components of BLC but may vary with respect to HLC, and the ‘non-native speaker’^2^ who will exhibit variability in both BLC and HLC. Attriting populations, however, are similar to late L2ers in that they diverge from monolingual control populations in both domains in their L1, indicating that even for native speakers, becoming bilingual will affect performance on skills belonging to both BLC and HLC.

#### Basic Language Cognition in L1 Attrition

There are a host of findings demonstrating that many of the language components which belong to BLC, and which are therefore assumed to show little variance among ‘native speakers,’ are subject to change and L1 attrition in immersed late bilinguals. These include^3^:

Accentedness: while monolingual populations are typically perceived to be at ceiling in global foreign accent rating experiments, several studies have established an increase in variance of such ratings in immersed late bilinguals which can lead to some attriters being perceived as unambiguously non-native (e.g., de Leeuw et al., 2010; Hopp and Schmid, 2013; Bergmann et al., 2016; Karayayla, 2018: ch. 4) and subtle shifts occurring in the production of both segmentals and suprasegmentals away from monolingual native norms (e.g., Mennen, 2004; de Leeuw, 2008; Chang, 2012; Bergmann et al., 2016).
Fluency: in both free and elicited discourse, L1At populations have consistently been demonstrated to be less fluent than monolinguals, as indicated by a slower speech rate and higher incidence of pauses, filled pauses, repetitions and self-corrections (e.g., Dostert, 2009; Schmid and Fägersten, 2010; Cherciov, 2011; Yılmaz and Schmid, 2012; Bergmann et al., 2015).
Lexical access: L1At populations are less productive at generating lexical items in Verbal Fluency tasks (e.g., Waas, 1996; Yagmur, 1997; Keijzer, 2007; Varga, 2012; Schmid and Jarvis, 2014) and slower and less accurate in naming tasks (e.g., Mägiste, 1992; Ammerlaan, 1996; Baus et al., 2008) than monolingual controls, suggesting that their access to even the highly frequent elements that are typically elicited in such tasks is delayed.
Overt/null pronouns: This is an example of grammatical features which form part of BLC. There are a large number of investigations demonstrating that overt pronouns come to be overgeneralised to contexts where monolingual natives would use null pronouns by attriters of pro-drop languages such as Bulgarian, Italian, Greek, and Spanish (e.g., Tsimpli et al., 2004; Domínguez, 2013; Genevska-Hanke, 2017).

#### Higher Language Cognition in L1 Attrition

Given that even monolingual native populations are assumed to be stratified with respect to HLC, it is hardly surprising that this variance increases under the cognitive demands of bilingualism. A wide range of studies have demonstrated this, for example, for complex and infrequent syntactic phenomena. For example, several studies of embedding structures in L1 Turkish have found attriters to diverge most from monolinguals on those types of embedding which are morphologically the most complex (i.e., involve the highest number of suffixations/transformations) and occur least frequently in free speech (e.g., Yagmur, 1997; Yılmaz, 2011; Karayayla, 2018). In a similar vein, attriters are consistently outperformed by non-attrited controls when it comes to the completion of complex written tasks (such as *C*-tests or cloze tests, e.g., Schmid and Dusseldorp, 2010; Cherciov, 2011; Varga, 2012; Kasparian, 2015).

In addition to distinguishing between BLC and HLC, Hulstijn’s model furthermore differentiates ‘core’ and ‘periphery’ aspects of language proficiency: core components refer largely to linguistic knowledge and the speed with which it can be processed, while peripheral components refer to the more metalinguistic skills, such as interactional ability and knowledge of the characteristics of different types of (spoken or written) discourse (Hulstijn, 2015, p. 41). The development of these HLC/peripheral skills in L1At has often been assessed based on the Can-Do Scales developed within the Common European Framework of Reference (see Hulstijn, 2015: ch. 10 for an in-depth discussion of the CEFR and its relationship to the BLC/HLC model, see below section “Study 1: Proficiency Measures Relating to Higher Language Cognition (HLC)” for details on the scales), and such studies tend to find larger differences between attriters and controls when it comes to reading and writing skills than with respect to speaking and listening (e.g., Opitz, 2011). This finding tentatively suggests that HLC components of language proficiency may be more vulnerable than BLC components – a hypothesis in need of further corroboration, but in line with the basic assumptions of the model.

#### Bilingual Development and the BLC/HLC Model

The findings presented above suggest that the BLC/HLC model can profitably be extended to include the development of language proficiency under conditions of L1At. The fact that the model assumes a global and holistic approach to defining language proficiency furthermore makes it ideally suited for an investigation of the relative development of proficiency in both the L1 and the L2 within the framework of language dominance, as the components of the model can be assessed and compared across languages. The findings presented above illustrate that, for linguistic features in both domains, attriting populations develop increased variability and diverge from the native baseline.

What remains entirely unclear, however, are the conditions or predictors which drive these changes: In all of the studies listed above, some of the attriters remain within the native range while others fall squarely outside it, but assessments of the impact of predictor variables have remained largely inconclusive (e.g., Schmid, 2011b). In other words, it is unclear to what extent external factors such as the frequency and domains of L1 and L2 use, the length of residence, or levels of attitude and motivation, contribute to the deterioration or maintenance of any particular linguistic feature. This paper attempts to address this knowledge gap by adopting an innovative approach that we believe is capable of assessing the development in both languages within an integrated framework. We will focus on those domains of language that allow us to make meaningful comparisons of the level of development in L1 and L2, namely measures related to lexical access and to the level of ability of performing tasks related to written language.

## Hypotheses and Research Questions

The present study sets out to test the hypothesis that the explanatory potential of investigations of language dominance can be enhanced by adopting a fully two-dimensional approach which takes into account performance in both L1 and L2. We furthermore assume that linear statistical models – that is, models based on regression slopes – may not be able to capture the complex interaction of different features of proficiency, personal background, exposure/use, and attitudes (henceforth: external factors), and propose that a classification into different types of language developers may allow a more detailed picture to emerge.

We ask the following research questions:

RQ1: What is the predictive power of external factors for measures of language proficiency in both the L1 and the L2 of long-term immersed bilingual populations in a multifactorial linear model?
RQ2: To what extent can the predictive power of external factors for measures of language proficiency in both the L1 and the L2 of long-term immersed bilingual populations be increased by adopting a data categorisation approach which does not rely on the linearity of relations?
RQ3: What are the differences between HLC and BLC aspects of proficiency with respect to RQs 1 and 2?

## The Study

### Ethical Approval

The data reported here were collected in 2004 (Study 1) when the PI (the first author of this paper) was affiliated with the Vrije Universiteit Amsterdam and in 2007 (Study 2) when both authors were affiliated with the University of Groningen. At this time, the humanities faculties at these institutions did not have a protocol for ethical approval nor an ethics committee, and there were no national guidelines in relation to this. All participants did provide written informed consent prior to the experiment. With hindsight we recognise the lack of formal ethical approval for the studies to be a shortcoming – which, unfortunately, cannot be addressed retrospectively. However, all of the materials and experiments reported on here have been used by both authors in subsequent investigations for which ethical approval was duly granted according to the protocols required by different institutions, including the University of Groningen, the University of Essex and the Humboldt University, Berlin. We are therefore convinced that the research design in itself is unproblematic from an ethical point of view.

### Participants and Predictor Variables

The data for the present study were collected from four experimental and three control populations. Study 1, which focuses on the development of Higher Language Cognition (HLC), was conducted with native German speakers (*n* = 106) with between 9 and 58 years of residence (LoR) in the Netherlands (*n* = 53, mean LoR 34.28) and the Greater Vancouver area, Canada (*n* = 53, mean LoR 37.09). Study 2, investigating aspects of Basic Language Cognition (BLC), was conducted with 87 migrants with between 10 and 43 years of residence in the Netherlands. 52 of these speakers were Turkish natives (mean LoR 22.57), while 35 were native speakers of Moroccan Arabic (mean LoR 23.31).

The experiments described hereunder investigate both the L1 and the L2 of these speakers. In Study 1, data collection was done in a single session, as the collection of L2 data was restricted to two tasks tapping into controlled and highly monitored language skills. Study 2, on the other hand, was conducted in two different sessions and by different researchers, due to considerations linked to language mode: Session 1 collected both experimental and informal spoken L1 data, while Session 2 (which took place several months later) collected similar data in the L2. In order to induce a predominantly monolingual language mode for these experiments, we considered it important that the researcher should be a speaker of the language which was the focus of the experimental session (Turkish/Moroccan Arabic in Session 1, Dutch in Session 2) with no knowledge of the other language. The researchers conducting Session 1 were recent arrivals to the Netherlands and native speakers of Turkish and Moroccan Arabic, respectively. Session 2 was conducted by two research assistants who were native speakers of Dutch but had no knowledge of either Turkish or any variety of Arabic. Unfortunately but inevitably this led to some participant loss, with data from only 63 participants available at Session 2. In this and the following sections, the dataset comprising 87 participants will be referred to as ‘Full dataset,’ while the dataset comprising the 63 participants with L2 data available will be referred to as ‘Limited dataset.’ **Table 1** summarises participant characteristics for both studies.

**Table 1:** Participant characteristics.

		Study 1		Study 2 (full dataset)		Study 2 (limited dataset)	
L1		German	German	Turkish	Moroccan Arabic	Turkish	Moroccan Arabic
L2		English	Dutch	Dutch	Dutch	Dutch	Dutch
*n*		53	53	52	35	46	17
Females *n* (%)		34 (61.2)	35 (66.0)	32 (61.5)	7 (20)	31 (67.4)	7 (21.5)
Age (range)		63.23 (37–88)	63.36 (37–85)	43.15 (28–61)	46.63 (30–65)	43.04 (28–61)	43.57 (31–65)
Age at emigration (range)		26.13 (14–47)	29.08 (16–51)	20.38 (14–42)	24.06 (18–32)	19.74 (14–42)	24.29 (18–32)
Length of residence (range)		37.09 (9–54)	34.28 (14–58)	22.15 (10–35)	22.57 (10–43)	22.62 (10–35)	19.29 (10–38)
Educational level (%)	Primary	13 (24.5)	9 (17.0)	17 (32.7)	10 (28.6)	16 (34.0)	4 (28.6)
	Secondary	27 (50.9)	27 (50.9)	10 (19.2)	22 (62.9)	8 (17.0)	11 (57.1)
	Tertiary	13 (24.5)	17 (32.1)	25 (48.1)	3 (8.6)	23 (48.9)	2 (14.3)


#### Language- and Attitude-Related Background Factors

Data on participants’ biography, language learning history, language use and language attitudes were collected by means of the same questionnaire in both studies^4^. The questionnaire comprises a total of 77 questions in different formats: open questions (e.g., birthplace, profession, personal reflections), Likert-scale questions (e.g., levels of use, attitudes and preferences), and interval questions (e.g., age). The questionnaire and its coding and analysis are described in detail in Schmid (2011a). The questionnaire was used by the researcher as the basis for a semi-structured interview, where the participants were prompted to talk about themselves, their biography and their languages, freely, informally and in detail (the procedure for conducting such an interview is described in Schmid, 2011a). All interviews were transcribed and coding was checked against both the recording and the notes taken during the session. The variables derived from this and used in the present study are described below, an overview of responses per question and group is presented in the **Supplementary Table S1**.

##### Self-reported language proficiency

All participants were asked to rate their proficiency in both their L1 and L2 (used here to refer to the language of the country in which they were living at the time of data collection) both at the time of migration and at the time of the interview, and also to state which of these languages they felt was the stronger at the present time. There were several interesting differences between groups, such that regardless of L1 background almost none of the migrants to the Netherlands knew more than a few words of Dutch before arrival while more than half of the migrants to Canada rated themselves as intermediate or proficient in English at arrival. At the time of testing, most of the bilinguals felt they had intermediate or good proficiency in the L2, with the English L2 speakers again standing out. The Germans rate their proficiency in Dutch more highly than the Turks and the Moroccans, possibly reflecting the advantage the close typological relatedness between their L1 and their L2 gives them. With very few exceptions, everyone rated their L1 proficiency at migration as ‘good’ or ‘very good,’ but that proportion dropped across the board for proficiency at the time of testing, although only one single speaker described it as ‘bad.’ Only among the L2 English speakers did more than a quarter of participants feel that their L2 had become stronger than their L1, while just over half of all L1 Germans thought both languages were equally good. Balanced bilingualism or dominance reversal were much rarer among the Turks and the Moroccans, with strong majorities in both groups feeling that their L1 remained their stronger language.

##### Language exposure and use

The questionnaire contains a total of 25 5-point Likert Scale questions on frequency of L1 exposure and use:

• L1 and L2 use within the family (with partner – 4 items, with children – 4 items, with grandchildren – 4 items)
• L1 and L2 use with friends and acquaintances (4 questions)
• L1 and L2 use at work (2 questions)
• Frequency of use of L1 media (radio, tv, newsmedia, books, 4 questions)
• Frequency of visits to the home country (1 question)
• Overall contact with and use of the L1 (2 questions)

Some of these items had to be excluded for the present analysis as variability was too low (for example, virtually all of the Netherlands-based participants stated that they visited their home country at least once a year). The general picture which emerged across these questions was that most participants continued to use their L1 on a fairly regular basis, the Germans slightly less so than the Turks/Moroccans. All groups had good social contacts within their new country but there was some variance, with roughly two-thirds of Turks and Moroccans reporting more friends who shared their L1 while over half of the Germans said their social network was composed mainly of native speakers of the L2. When it comes to language use in the family, the Turks stand out somewhat from the other groups with a much stronger claimed adherence to an L1-only policy, while half of the Germans report using L2 only (many people noted in the interview that they would have liked to have persisted more on using their L1 with their children, but that they had faced too much resistance and had given up). The Moroccans appear to occupy an intermediate position. A similar picture appears across most language exposure and use questions: most participants appear to have a fairly clear preference for one language in each context, with the Turks and Moroccans leaning more toward the L1 than the Germans. The only exception to this is the use of the native language for professional purposes, which stands at around 20% for both German groups but is quite rare for the Moroccans and Turks.

##### Attitudes

Where attitudes toward the native language are concerned, the views seem more homogenous across groups, with over 75% in all groups saying it is important or very important to them to maintain their L1 and almost the same proportion of respondents saying it is important to them to pass it on to their children. An interesting finding emerged from the question “Which language do you prefer?”: while the Germans in Canada were split roughly evenly between their L1 and their L2, and three quarters of the Turks and Moroccans stated a preference for their L1, the only group that had a substantial proportion of self-reported balanced bilinguals for this questions (that is, of speakers who report ‘no preference’) were the Germans in the Netherlands. This suggests that the similarity between L1 and L2 for the German–Dutch bilinguals may have facilitated the perception of a more balanced bilingualism. Interestingly, while there was only one speaker who reported ‘no preference’ on this question in the German–Canadian group, over 50% responded in the affirmative when asked whether they felt they were balanced bilinguals, while for all other groups, the answers across both questions seemed to be largely consistent.

##### Principal component analysis

The overview of findings presented here points to two general problems concerning personal background data in language attrition research. Firstly, there are many questions with potentially important information for which there is missing data from a substantial proportion of informants – for example, not all participants have a partner and/or children. Secondly, as pointed out above (see section “Predictor Variables in Bilingual Development”), the data are not normally distributed: for most of the variables reported here, there is either a skewness toward the L1 end of the scale or a bimodal distribution. Both phenomena are a natural and inevitable characteristic of attrited populations: most studies find sustained preference for the native language, particularly where it comes to self-assessed proficiency. Furthermore, as is predicted by the Complementarity Principle (Grosjean, 1997, 2016), most people tend to prefer one or the other language across most domains.

In order to alleviate these problems as well as reduce the number of predictor variables for analysis, we conducted a Principal Component Analysis (PCA). We addressed the problem of missing values by replacing them in each case with the neutral point on the scale. We chose this strategy over the more common approach of imputing missing values based on the rationale that, for the data in question, values either above or below the neutral measure would (incorrectly) suggest that the relevant language (the L2 if the imputed value was below the mean and the L1 if it was above it) played a role in the prediction of the outcome variables while setting it to neutral allowed the case to be included in the analyses without such an effect.

All variables were standardised to the same scale prior to entry into the PCA, with the maximum value in the dataset (e.g., 88 in the case of *age at testing*) set to 1 and the minimum value set to 0. The PCA (Varimax Rotation, extraction of factors with *Eigenvalues* > 1.000) identified a total of six components which were saved as factors (see **Supplementary Table S2** for the full component matrix). The first component comprised 9 factors relating largely to the frequency of casual and informal use of the L1 and the L2, that is, with family and friends (Cronbach’s α = 0.881) and was labelled *Interactive Use*. The second component, *Personal Background*, comprised the variables age, length of residence and education (α = 0.609). The third component related to *Perception* and comprised the answers to the questions about current L1 proficiency and whether that had changed since immigration (α = 0.455). Component 4 comprised the *Attitude*-related variables of importance to maintain the L1, transmit it to the children, and culture of preference, alongside the frequency of use of L1 media (books, TV, and radio) (α = 0.627). Overall *Contact* with the L1 was a unifactorial component, while *ProfessionalUse* of both L1 and L2 made up the last component (α = 0.476).

The first four components were normally distributed. There was a slight negative skew for *Contact* [*D*(190) = 0.091, *p* = 0.001] and a more pronounced positive one for *ProfessionalUse* [*D*(190) = 0.116, *p* < 0.001]. These variables were log-transformed after a constant was added to make all values positive and the scale for the negatively skewed component was inverted. The transformed components were no longer skewed [Contact: *D*(190) = 0.049, *p* = 0.2; Professional Use: *D*(190) = 0.061, *p* = 0.086].

#### Outcome Variables

The data collection for both studies included a native language control group for the L1 tasks (German in Study 1, Turkish and Moroccan Arabic in Study 2), and Study 2 also used a Dutch native control group for the L2 tasks. Controls were matched with the relevant experimental populations for age, gender, educational background and, in Study 2, region of origin within the L1 country. It is not the purpose of the present investigation to probe into issues of general proficiency or overall attainment against an idealised monolingual baseline, but to assess to what degree development has taken place in both languages within the proficiency space defined by the performance of the immersed bilingual population. While the descriptive statistics given below include the results from the control group as indicative values, they were therefore not used in the inferential statistics.

##### Study 1: proficiency measures relating to higher language cognition (HLC)

In Study 1, participants completed four tasks^5^: a *C*-Test and a detailed self-assessment, each in both their L1 and their L2. Each of the two *C*-Tests comprised five short texts with a total number of 100 gaps determined by the schema proposed by Grotjahn (2010), and each correctly filled gap was awarded one point, so the maximum possible score in each language was 100. The self-assessments contained 43 5-point Likert-Scale items for the subdomains Listening (8 items), Reading (7 items), Speaking (17 items), and Writing (11 items). These items were constructed based on the ALTE Can-Do statements for levels C1 and C2 of the Common European Framework of Reference for Languages (CEFR, see Hulstijn, 2015: ch. 10). Responses were coded from 1 (“I cannot do this”) to 5 (“I can do this without any difficulty”). Averages were created for the subscales as well as globally, with the maximum possible score being 5 and the minimum being 1.

Since the *C*-Test in the second language was different for the two populations (one being in English, the other in Dutch) the results were standardised for both groups by setting the lowest score in either population to 0 and the highest to 100. None of the tasks differed significantly across populations, although the Can-Do scales for the L1 approached significance, with the Dutch L2 speakers rating themselves somewhat higher than the English L1 speakers. The results are summarised in **Table 2**.

**Table 2:** Outcome variables, German L1 group.

	Germans in Canada (GECA)		Germans in the Netherlands (GENL)		German controls (GECG)	

	Mean (std)	Range	Mean (std)	Range	Mean (std)	Range
*C*-Test L1	75.26 (11.61)	46–95	77.21 (13.81)	38–95	82.21 (8.90)	59–99
Can-Do L1	3.92 (0.59)	2.63–4.67	4.12 (0.46)	2.71–4.67	3.83 (0.5)	2.76–4.65
*C*-Test L2 original scores	70.42 (17.82)	15–92	76.31 (17.33)	12–99		
*C*-Test L2 standardised	71.98 (23.14)	0–100	73.92 (19.92)	0–100		
Can-Do L2	3.99 (0.61)	2.42–4.67	3.81 (0.68)	1.80–4.78		


Tests of normality (K–S, bilingual data only) were significant at *p* < 0.01 for all four variables, and visual inspection revealed all of them to be negatively skewed. The variables were therefore inverted and log-transformed, which resolved the normality issue for all except the L2 *C*-Test. That variable was root-transformed instead, resulting in a normal distribution. All variables were subsequently re-calculated to the same scale, so that in all cases the lowest score achieved was set to 0 and the highest to 1. The resulting standardised scores all correlated with the original scores above 0.95 (all *p*’s < 0.001). Lastly, we calculated an average score for both tasks in the L1 as well as in the L2 (both were normally distributed, with a lower bound of the significance at 0.2).

##### Study 2: proficiency measures relating to basic language cognition (BLC)

Study 2 also used four instruments: Firstly, there were two Picture Naming Tasks (one in the L1, Turkish or Moroccan Arabic, and one in the L2, Dutch) in which participants were asked to say aloud the name of 78 objects which they saw as line drawings on a computer screen. The pictures were selected from the Snodgrass and Vanderwart (1980) dataset and controlled for cultural appropriateness, cognate status, item frequency and semantic and phonological relatedness between consecutive items (see Yılmaz, 2013 for details). Presentation was done through E-Prime 1.0 with a Serial Response Box and microphone to collect RTs, and all experiments were audio-recorded for later checking and verification of accuracy. Data were trimmed by eliminating all RTs below 250 ms as well as all items with inaccurate or missing responses. Outliers were defined as RTs higher than the mean plus two standard deviations, and these values were reduced down to the threshold for outliers. Based on these measures, the average RT for each participant in the L1 and in the L2 was calculated (see **Table 3**).

**Table 3:** Response times (ms) Picture Naming Task in L1 and L2.

		PNT L1	PNT Dutch
Turks in the Netherlands (TRNL)	Mean	1,123	1,293
	Std	154	146
	Range	805–1454	1,000–1,601
Turkish controls (TRCG)	Mean	1,110	
	Std	136	
	Range	855–1442	
Moroccans in the Netherlands (MANL)	Mean	1,068	1,120
	Std	175	259
	Range	689–1400	747–1482
Moroccan controls	Mean	956	
	Std	155	
	Range	720–1284	
Dutch controls	Mean		895
	Std		121
	Range		620–1198


In this case, the L1 naming latencies of the two bilingual groups did not differ substantially from each other. Nevertheless, in order to ensure that language-specific differences would not impact on the results, we followed the same strategy for standardisation within the language groups as described above under Study 1 (based on the Full Dataset). L2 naming latencies were standardised only on the basis of the bilingual data and did not include the monolingual data.

The second set of variables was derived from a semi-structured interview conducted by a native speaker of the language in question (Turkish or Moroccan Arabic in Session 1, Dutch in Session 2) with no knowledge of the other language. Both interviews were autobiographical, informal, and focused on different aspects of the emigration experience. All interviews were transcribed and coded according to the guidelines set out in the CHILDES project for the CHAT format (MacWhinney, 2000). The following variables were subsequently extracted from these transcriptions:

• VOCD (McKee et al., 2000). For the Turkish and Dutch data, VOCD was based on the lemmatised transcriptions by restricting the analysis to the %mor-tier. However, as there are no mor-grammars available for Moroccan Arabic, VOCD was calculated based on the main participant tier. In order to compensate for both the effect of language and the different methods of data extraction, VOCD in L1 was standardised separately for each speaker population. For Dutch VOCDs standardised scores were calculated based on all bilingual speakers (i.e., both Turkish and Moroccan participants).
• Fluency: for each participant, the number of filled pauses, repetitions and retractions (self-corrections) was counted and subsequently standardised to incidence per 1,000 tokens. Fluency variables were standardised across the groups for all speakers in the same way as described for the other variables above and averaged to one overall fluency measure per speaker in L1 and L2.

Tests of normality (K–S) showed no deviations from the normality assumption for any of the above variables. All were standardised to the same scale and direction (0 being the worst attained score and 1 being the best), and subsequently, one average measure for L1 and L2, respectively, was created by averaging the three subtasks.

## Results

In order to assess to what extent the variables established above for personal background, use of L1 and L2, and attitudes may be used to predict the outcome variables we conducted two analyses for each dataset: a multivariate analysis of covariance (MANCOVA), which creates an overall model but also allows identifying the regression slopes associated with each predictor for each outcome variable (RQ1), and a discriminant function analysis (DA), which allows a non-linear assessment of the impact of the predictors (RQ2). Each analysis was conducted separately for HLC aspects of proficiency (Study 1) and BLC aspects of proficiency (Study 2) (RQ3).

### MANCOVAs

#### Study 1: HLC Aspects of Proficiency

The first MANCOVA was conducted on the four standardised outcome variables from Study 1: *C*-Test L1, *C*-Test L2, Can-Do L1 and Can-Do L2. The six components identified by the PCA were entered as covariates. All components were entered together. Roy’s Largest Root was significant for all components except *Contact* (see **Table 4** for the full results). *Interactive Use* was significantly associated with both of the L2 measures, with a higher level of L1 use associated with a lower L2 *C*-Test score and L2 self-rating, but did not influence outcomes in the L1. The *Personal Background* component was associated with both self-assessments, a higher score on this component (reflecting higher age, longer length of residence and a lower educational level) leading to participants rating themselves better in L1 and poorer in L2 – without, however, this being reflected in the more objective proficiency scores yielded by the *C*-Test. *Perception* influenced both L1 measures, with participants who claimed their L1 had not changed and was at a high level at the present time achieving better scores on the *C*-Test and rating their proficiency more positively. *Attitude* had the most consistent effect: participants for whom the maintenance of their L1 and its transmission to the next generation was more important and who stated a preference for their home culture achieved better scores on the L1 *C*-Test and also rated themselves more positively in both languages. A higher level of use of the L1 for professional purposes *and* a lower level of use of the L2 was associated with higher C-Test scores in both languages. For the full models, effect sizes were medium to large, partial η^2^ values ranging from 0.22 to 0.33, while the individual associations were weak at best with partial η^2^s between 0.05 and 0.2.

**Table 4:** MANCOVA for HLC proficiency tasks in Study 1.

	GLM				L1				L2			

					*C*-Test L1		CanDo L1		*C*-Test L2		CanDo L2	

	Roy’s	
	largest	
	root	*F*(4,89)	*p*	Partial η^2^	*p*	Partial η^2^	p	Partial η^2^	*p*	Partial η^2^	*p*	Partial η^2^
Interactive use	0.446	9.925	<0.001	0.304^∗∗∗^					<0.001	0.126^∗∗∗^	<0.001	0.154^∗∗∗^
Personal background	0.526	11.693	<0.001	0.333^∗∗∗^			<0.001	0.076^∗∗∗^			<0.001	0.099^∗∗∗^
Perception	0.322	7.164	<0.001	0.248^∗∗∗^	<0.001	0.140^∗∗∗^	<0.01	0.096^∗∗^				
Attitude	0.332	7.388	<0.001	0.250^∗∗∗^	<0.01	0.108^∗∗^	<0.001	0.177^∗∗∗^			<0.05	0.048^∗^
Professional use	0.293	6.509	<0.001	0.220^∗∗∗^	<0.001	0.211^∗∗∗^			<0.01	0.104^∗∗^		
Contact	0.028	0.613	0.654	0.027								


*^∗^*p* < 0.05, ^∗∗^*p* < 0.01, and ^∗∗∗^*p* < 0.001.*

In order to assess to what extent including both languages in the model improves explanatory validity (RQ2), we repeated the MANCOVA twice. The first model included only the L1 measures as dependent variables, and the second only the L2 measures. Here, *Interactive Use* and *Personal Background* variables only became significant for the L2, while *Perception* was significant only for the L1. *Attitude* and *Professional Use* were significant predictors in both models, while *Contact* was not significant for either language. Except for the impact of professional L1 use on the L1, effect sizes were considerably lower than in the full model, ranging from 0.06 to 0.22 (see **Supplementary Table S3**).

#### Study 2: BLC Aspects of Proficiency

The findings were much less revealing for the less controlled aspects of language use tested in Study 2. Here, the only component that yielded a significant result overall was *Interactive Use*, with a higher level of L1 use in informal contexts associated with slightly slower responses on the L1 PNT as well as a reduction in L1 disfluencies. Partial η^2^s were around 0.11 (weak effect) for the individual measures and 0.27 (medium effect) for the overall model. A few other significant relationships were observed (see **Table 5**), but in those cases the overall model was not significant. These findings confirm earlier studies showing that accounting for informal features of language attrition on the basis of background variables is highly problematic in models based on linear regression slopes (e.g., Schmid and Dusseldorp, 2010).

**Table 5:** MANCOVA for BLC proficiency tasks in Study 2.

	GLM				L1						L2					

					RT		VOCD		Fluency		RT		VOCD		Fluency	

	Roy’s	
	largest	
	root	*F*(6,43)	*p*	η^2^	*p*	η^2^	*p*	η^2^	*p*	η^2^	*p*	η^2^	*p*	η^2^	*p*	η^2^
Interactive use	0.369	2.648	<0.05	0.270^∗^	<0.05	0.115^∗^			<0.05	0.107^∗^						
Personal background	0.103	0.736	0.623	0.093												
Perception	0.236	1.69	0.147	0.191	<0.05	0.113^∗^									<0.05	0.113^∗^
Attitude	0.216	1.552	0.185	0.178			0.051	0.077	<0.05	0.107^∗^						
Professional use	0.095	0.678	0.668	0.086												
Contact	0.159	1.137	0.357	0.137			0.079	0.063								


*^∗^*p* < 0.05, ^∗∗^*p* < 0.01, and ^∗∗∗^*p* < 0.001.*

Repeating the MANCOVA for each language separately did not return a significant result for any of the predictors except *Attitude*, which was only significant in the L2 model (*p* < 0.05; partial η^2^ = 0.155).

The findings from these two analyses are interesting in the light of previous investigations of L1 attrition in that they underscore that, while including results relating to performance in both languages can increase explanatory adequacy, analyses looking for linear regression slopes typically yield few results and have little predictive power. While the analyses of the HLC skills presented above are overall significant, the predictive power of the independent variables is limited and inconsistent, and effect sizes are weak. The situation is even worse with respect to BLC aspects of proficiency, where no coherent picture emerges at all.

### Discriminant Function Analysis (DA)

The very limited explanatory value of the two General Linear Model analyses described above points to a fundamental problem in research on bilingual development: the most common statistical analyses, such as regression or ANOVA, are only able to capture *linear* trends and correspondences in the data (i.e., correlation coefficients or regression slopes). In other words, any one predictor will only be revealed as significant if its impact on the outcome variable is the same for all or most of the participants. This can be seen, for example, in the fact that *Professional L1 Use* has the strongest impact on formal tasks such as the *C*-Test: it makes sense that individuals who engage with language as part of their job would develop enhanced awareness of style, orthography etc., facilitating these kinds of tasks. However, this relationship may not hold for all speakers (e.g., some speakers may retain excellent skills despite not ever using their L1 professionally) and may be far more complex for other types of background variables. For example, it is possible that some factors may interact with each other in non-linear ways (this has been suggested, for example, for length of residence and L1 use, de Bot et al., 1991; Schmid, 2011b).

**FIGURE 2:**
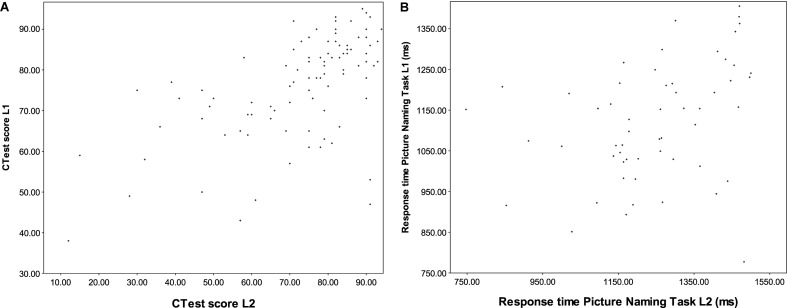
**(A)** The relationship between C-Test scores in L1 and L2 in Study 1. **(B)** The relationship between PNT naming latencies in Study 1 and Study 2.

A more serious problem for linear analyses is the fact that, as pointed out above (see section “Investigations of Development in Both Languages”) the balance between the L1 and the L2 is not the same for all members of the population: some speakers may preserve excellent proficiency in the L1 while also excelling in the L2, while for others, the development or maintenance of one language may come at the expense of the other, and others still may regress in their L1 without ever reaching very advanced levels of the L2. This variance cannot be captured by single-dimensional approaches, where the score obtained in one language is subtracted from that of the other, but it also eludes analysis by linear modelling. Consider the scores obtained on the *C*-Test in Study 1, and on the PNT in Study 2. For the former, there is a moderate positive correlation (*r* = 0.617, *p* < 0.001), indicating that participants who perform well in one language tend to perform well in the other (as pointed out above, this is probably due to the fact that general awareness of formal constraints facilitates this kind of task in any language), but there are quite a few marked exceptions to this trend, as visualised in **Figure 2A**: for example, the two crosses toward the bottom right of the panel represent two participants who are among the lowest performers in the L1 but score within the top 15% in the L2. For the less controlled aspects of language use belonging to BLC aspects of proficiency, such as lexical access measured by the PNT and VOCD, on the other hand, no significant correlation between languages obtains, and no overall pattern can be detected from the scatterplot (**Figure 2B**).

**FIGURE 3:**
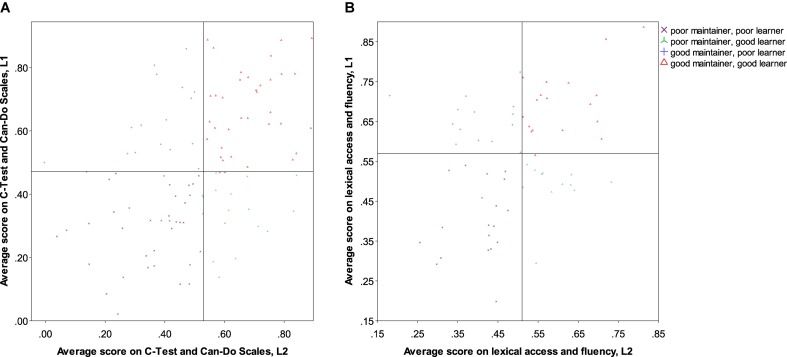
**(A)** Categorisation according to median split of averaged proficiency measure for participants in Study 1. **(B)** Categorisation according to median split of averaged proficiency measure for participants in Study 2.

In order to account for the four different types of bilingual balance identified in Treffers-Daller’s (2016, p. 261) ‘typology of language dominance based on language proficiency,’ analyses are therefore necessary that do not assume (negative or positive) linear relationships between development and proficiency in both languages. We propose a method here that proceeds from a *median split* of all participants in Study 1 and in Study 2. This was calculated on the basis of a single average score per language for the standardised variables *C*-Test and Can-Do Scales in Study 1 and PNT, VOCD and fluency in Study 2. Each participant was then categorised as having scored either above or below the median in each language, yielding four groups (see **Table 6**). The division is visualised in **Figure 3A** (Study 1) and **Figure 3B** (Study 2).

**Table 6:** Subpopulations of bilinguals based on median split of proficiency scores.

	Study 1		Study 2	

	*n*	%	*n*	%
Good maintainer, good learner (performance above median in both languages)	36	34	18	28.6
Good maintainer, poor learner (performance above median in L1, below median in L2)	17	16	14	22.2
Poor maintainer, good learner (performance below median in L1, above median in L2)	17	16	12	19.0
Poor maintainer, poor learner (performance below median in both languages)	36	34	19	30.2


The four-level categorical variables thus obtained for Study 1 and Study 2, respectively, were subsequently used as the grouping variable in two Discriminant Function Analyses (DA). DA attempts to find the best combination of predictors based on which as many cases as possible can be classified into the predetermined categories (Huberty and Olejnik, 2006). It is useful for the present investigation because (a) category membership is a nominal variable and thus does not imply any form of linear relationship or ranking and (b) the number of predictors is not limited based on the number of observations, as is the case, for example, in regression analyses and (M)ANCOVAs. The same 21 variables that were used for the Principal Component Analysis described above (see section “Principal Component Analysis”) were entered as predictors into the DA.

For Study 1, three functions were identified which together significantly discriminated the four groups [Wilks’ λ = 0.212, χ^2^(63) = 143.297, *p* < 0.001]. The first function explained 54.6% of the variance (canonical *R*^2^ = 0.54), the second explained 33.7% (canonical *R*^2^ = 0.42) while the third explained only 11.7% (canonical *R*^2^ = 0.20). The cutoff point for factor loadings was set at 0.3 (the same threshold as used for the PCA).

**FIGURE 4:**
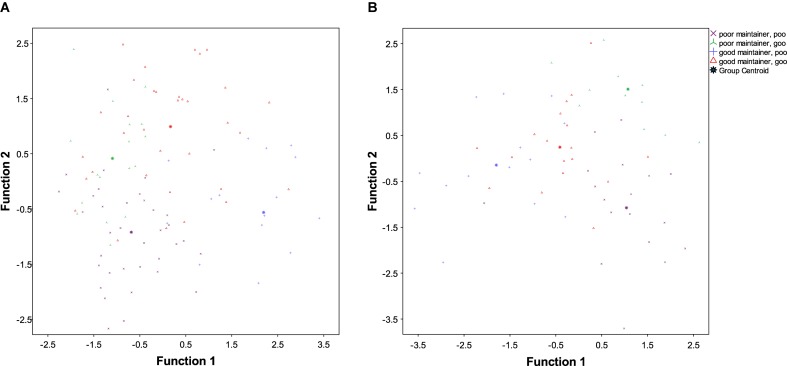
**(A)** Canonical discriminant functions, Study 1. **(B)** Canonical discriminant functions, Study 2.

Function 1: The factors loading on the first function mainly related to overall, interactive and informal use: the strongest factor here was the use of the L1 within the family, while the use of the L2 with friends and at work loaded negatively on this factor. The length of emigration was also a negatively loaded factor. An inspection of the group centroids (**Table 7**) suggests that this function was mainly associated with the maintenance of the L1: irrespective of their level of success in the L2, good maintainters tended to score positively on this function (that is, to have comparatively high levels of use of the L1 and low levels of use of the L2 in the contexts listed above, and short periods of residence), while poor maintainers scored negatively. This tendency is illustrated in **Figure 4A**, which also reveals it to be more pronounced for the poor maintainers: while a number of the good maintainers/good learners score in the negative space of Function 1, none of the poor maintainers fall into the positive half of the chart, and only very few of the good maintainers/poor learners fall into the negative one. This suggests that good maintainers/good learners may possess high levels of aptitude, which allow them to attain high levels of proficiency in the L2 and overcome the negative impact of low levels of L1 use, high levels of L2 use and/or long periods of residence, retaining high levels of proficiency in the L1.

**Table 7:** Discriminant Analysis Study 1, Functions at group centroids.

	Function 1	Function 2	Function 3
Poor maintainer, poor learner	-0.684	-0.922	0.277
Poor maintainer, good learner	-1.096	0.415	-0.976
Good maintainer, poor learner	2.195	-0.559	-0.362
Good maintainer, good learner	0.165	0.99	0.355


Function 2: The second function mainly differentiates good vs. poor learners (see **Figure 4A**), although there also seemed to be some impact on maintenance in that both among the good and among the poor learners the good maintainers scored more highly than the poor maintainers. The strongest factor in this function was educational level. Somewhat surprisingly, the use of the L1 for professional purposes also seemed to have a positive impact on L2 acquisition as well as maintenance. This suggests that using the first language professionally may help develop a higher sensitivity to some linguistic properties which are helpful in acquiring L2 proficiency, in particular as concerns the relatively controlled and monitored HLC skills measured in Study 1. Lastly, self-evaluated proficiency in the L2 also loaded on this factor.
Function 3: The last function was mainly associated with attitudinal factors. Variables loading onto this function were self-perceived changes to the L1 and current proficiency in the L1, the language and culture of preference and the proportion of friends with German as their L1. Interestingly, this function seemed to be associated with balanced vs. unbalanced bilingualism: those bilinguals who scored low or high in both their languages had a higher score on this function than those for whom one language was stronger (good maintainers/good learners scoring somewhat higher than poor maintainers/poor learners). The group of poor maintainers and good learners had the lowest score on this function, followed by the good maintainers and poor learners. This tentatively suggests that a positive attitude toward the native language may support the development of the L2 toward its full capacity, matching that attained in the L1.

Based on these three functions, the DA was able to accurately classify 70.8% of original cases. In other words, 70.8% of all participants were assigned to the same of the four groups listed above by the DA and by the median split (see **Supplementary Table S4**).

The DA for Study 2 also identified three functions which together significantly discriminated the four groups [Wilks’ λ = 0.143, χ^2^(63) = 90.376, *p* < 0.05]. The first function explained 48.6% of the variance (canonical *R*^2^ = 0.58), the second explained 34.1% (canonical *R*^2^ = 0.49) while the third explained 17.3% (canonical *R*^2^ = 0.33). Like in Study 1, Function 1 distinguished good and poor maintainers, while Function 2 distinguished good and poor learners (see **Table 8** and **Figure 4B**).

**Table 8:** Discriminant Analysis Study 2, Functions at group centroids.

	Function 1	Function 2	Function 3
Poor maintainer, poor learner	-0.333	0.093	1.012
Poor maintainer, good learner	-1.692	0.299	-0.664
Good maintainer, poor learner	1.481	1.358	-0.342
Good maintainer, good learner	0.745	-1.385	-0.301

Function 1 comprised the language of preference, the language used with friends and the self-evaluated proficiency in the L2. As in Study 1, this function seemed mainly related to success in L1 maintenance, suggesting that people who preferred the L1, used the L2 less with friends and estimated their L2 proficiency lower were better maintainers (see **Figure 4B**).
Function 2 was again positively associated with level of education but also with self-assessed proficiency in the L1, and discriminated good and poor learners. Interestingly, good learners have a higher estimate of their own L1 proficiency than poor learners. Several other factors linked to the frequency of use of the L1 were positively associated with this function, but fell below the.3 threshold.
Function 3 comprised professional use of either language, with good maintainers/good learners reporting the highest levels here and poor maintainers/poor learners the lowest, while the unbalanced groups had an intermediate position. This suggests that professional interactions – irrespective of the language in which they take place – may be beneficial for overall language development, possibly through the addition of a distinct domain for language use.

Together, these three functions accurately predicted group membership in 76.7% of original cases (see **Supplementary Table S5**).

## Discussion

With the analyses presented above we have attempted to break new ground for the study of L1 attrition and language dominance. The knowledge gap we have addressed relates to the role of predictors in L1 attrition and the fact that, at the current state of knowledge, the empirical base for explanatory models of variability in L1 proficiency among immersed bilinguals is extremely weak. In other words, while we know that some individual speakers have attrited to a far higher degree than others, we do not know why. We therefore attempted to assess what circumstances in the environment of a particular speaker will facilitate the attrition vs. the maintenance of the L1. In order to do this, we adopted a novel approach. This proceeded from the assumption that explanatory models of language development, based on predictors comprising personal background factors as well as measures relating to exposure, use and attitudes, would be more powerful and more enlightening when both of the languages of the populations under investigation are taken into account.

In order to do this, we conducted two studies. The first one used linguistic measures related to Higher Language Cognition (mainly measuring participants’ ability to manipulate language in ways that are not part of spoken daily interaction, through performance on a *C*-Test and self-ratings of language skills in a range of domains), while the second investigated the development of Basic Language Cognition, in particular in relationship to lexical access (Hulstijn, 2015). Both studies assessed these measures in both the participants’ L1 (in Study 1, this was German, in Study 2 it was Turkish and Moroccan Arabic) and their L2 (Study 1: English and Dutch, Study 2: Dutch).

The first analysis attempted to identify linear relationships between the outcome measures on the one hand and the predictors on the other. It was demonstrated that including dependent variables relating to proficiency in both languages can considerably improve the explanatory validity of such models. With respect to the formal tasks relating to HLC proficiency measured in Study1, our results showed an impact of frequency of L1 use only for the L2 tasks, while aspects of personal background (such as age, education and length of residence) and introspective measures of proficiency and attitudes seemed to be reflected mainly in the self-ratings elicited by the Can-Do Scales in both languages. In the second analysis, findings were even more scarce and only suggested that higher levels of L1 use might have a facilitating – albeit very weak – effect on Reaction Times in lexical naming and fluency in informal speech in the L1.

The somewhat disappointing results from this analysis are fully in line with previous work on L1 attrition: as was pointed out above, few studies have been able to identify any consistent impact, let alone any strong explanatory power, of predictors on actual measures of L1 proficiency and performance.

For our second analysis, we therefore adopted a different approach. Firstly, we combined the different measures of proficiency (two measures per language in Study 1, three in Study 2) into one compound measure. This was done following Opitz (2011, 2013) who showed that group differences which are masked in analyses based on single tasks may emerge when a compound measure is created. Secondly, we classified each participant into one of four quadrants of the proficiency space, based on whether they had performed above or below the median in each of their languages. This resulted in the creation of four distinct types of developers: good maintainers/good learners, good maintainers/poor learners, poor maintainers/good learners and poor maintainers/poor learners.

We fully acknowledge that this classification suffers from a number of problems that categorisation of interval data invariably entails: firstly, there is substantial loss of variance incurred by collapsing all of these different scores into just four categories. Secondly and relatedly, it results in the classification of those cases who are closest to the (arbitrary) threshold established for the cutoff into one group, even though they are far more similar to individuals on the other side of the threshold in another group than to many cases in their own category. This becomes evident from the visualisation of the categorisation in **Figures 3A,B**, above: the area in the middle of each chart contains participants whose scores in both languages are very close to each other, but who were assigned to different groups. We feel, however, that the benefits of capturing a relationship between the two languages that may go hand in hand for some participants but be orthogonal for others outweigh these drawbacks, but we would be delighted to learn of other analyses that are able to achieve this without resorting to categorisation of data.

Given the lack of previous insights into what factors may predict the development of a native language in immersed bilinguals, the insights gained from this classification can only be described as both unexpected and dramatic. Our hypothesis that treating language proficiency as a two-dimensional construct was confirmed by the Discriminant Analysis which was able, in both studies, to classify around three quarters of all participants accurately. Given the substantial differences between the two studies, both in terms of the population and of the linguistic skills analysed, it was particularly striking that, in both cases, the first – and hence most powerful – of the three functions identified in the analysis related to L1 maintenance (irrespective of attained level of L2 proficiency) and comprised mainly measures related to informal language use: in both studies, those participants who had higher levels of language maintenance were the ones who used the L2 less with their friends and the L1 more with their family. This finding lends support to the often intuitively held view that more informal use of the L1 should be conducive to L1 maintenance – which, however, so far has lacked empirical substantiation. For example, a study on L1 attrition of lexical access and fluency measures very similar to the ones investigated in Study 2 here and using the same set of predictors (Schmid and Jarvis, 2014) finds no impact whatsoever of any factors linked to exposure. In a similar vein, a multivariate analysis of measures similar to the ones used in both studies here is presented by Schmid and Dusseldorp (2010). In the absence of the dimension presented by the measures in the L2, they conclude that “[l]anguage use in the more informal settings appears to have very limited protective function with respect to L1 attrition” (p. 152; see Schmid, forthcoming for a review of research attempting to link L1 use and L1 attrition).

Interestingly, the first function returned by the DAs, while successfully separating good and poor maintainers, seemed unrelated in both studies with success obtained in an L2, suggesting that the amount of informal use a participant makes of both her languages does not play a strong role when it comes to the development of either HLC or BLC skills in a second language. Here, it was found across both studies that the level of education as well as the level of self-perceived proficiency seemed to play a role. In Study 1, this function also affected L1 maintenance to some extent, suggesting that, when it comes to HLC, a higher level of education may be beneficial not only for L2 acquisition but also for L1 maintenance. Similarly, in this study, the use of the L1 at work was important for both successful acquisition and successful maintenance. In Study 2, the effects of these factors were less pronounced, which is hardly surprising given that the BLC skills investigated in this study are probably much less amenable to educational levels. A rather puzzling finding is that the strongest contributing factor here was self-assessed L1 proficiency, with higher levels of proficiency being associated with better L2 skills – again, this may be related to the (unassessed) individual difference of language learning aptitude, which may have facilitated both L2 acquisition and L1 maintenance.

The last function was the only one for which there was no common pattern across the two studies. In Study 1, it seemed that a more positive attitude toward the native language and its maintenance would contribute to a more balanced pattern of language dominance, while in study 2 it seemed that using either the L1 or the L2 professionally may facilitate a higher level of proficiency in both languages.

**FIGURE 5:**
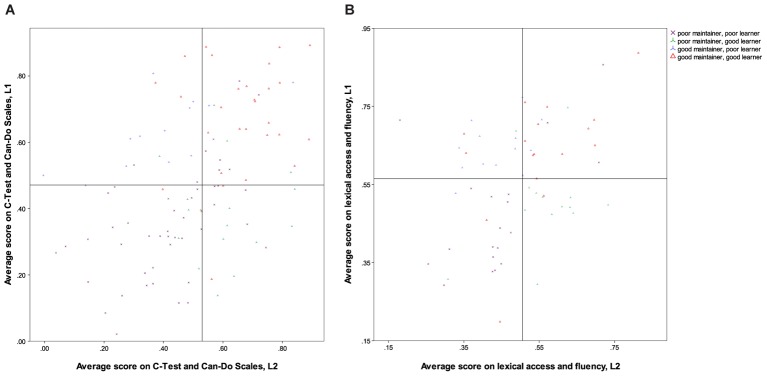
**(A)** Predicted group membership for Study 1. **(B)** Predicted group membership for Study 2.

It thus seems that the complex interaction of the predictors of both L1 attrition and L2 acquisition can be captured better by analyses which (a) plot out their results in a fully two-dimensional fashion and (b) do not rely on sweeping averages of the many necessary predictors, as we did through the Principal Component Analysis which yielded the independent variables used in the first set of analyses (MANCOVAs). As we pointed out above, the categorisation of data has a number of undesirable results, as it assigns cases which are very similar to each other to different groups. However, a closer look at the classifications yielded by the DA suggests that the negative impact of this may be less dramatic than one might have thought: **Figures 5A,B** depicts the median split division that was shown in **Figure 3** above. However, in this case, the markers showing the position of each individual do not represent their original group membership, but the group assigned to them by the DA.

In both studies, it is striking that, with few exceptions, most of the misclassified cases occur quite close to the median split lines. Recall that the DA does not have access to the actual scores of any of the individuals, only to the categorical group membership data. The fact that, even so, of the roughly 25% of misclassified individuals the vast majority are to be found among the more marginal cases underscores the potential value of such an analysis.

In order to gain more insight into the mechanisms of the development of language dominance, it may then be beneficial to adopt the approach suggested by Opitz, and scrutinise the more extreme cases of misclassification. We would like to illustrate this approach with the example of one individual participant in Study 2. This speaker attained the second highest score in the L1 and joint second highest in the L2, but the DA predicted her to be a poor maintainer and poor learner (represented by the purple cross toward the top right of the panel in **Figure 5B**). The participant in question is a Turkish woman who had come to the Netherlands aged 18 and, at the time of the data collection, had been living there for 27 years. While she used the L1 almost exclusively in her social life, she had in the early years of her emigration had contact with some Dutch women who had begun to teach her that language. She found that she very much enjoyed learning the language and, just before the time of data collection, had begun taking Dutch lessons for the first time (at the suggestion of her line manager). In the interview she talks about discovering aspects of the Dutch language and grammar that she had not previously been aware of, and what an enlightening and enjoyable experience this was for her. Furthermore, developing her Dutch skills also proved an empowering experience which changed her relationship to her overbearing and somewhat authoritative husband. It thus seems that, for this participant, a number of factors not measured in the present study, but probably relating to a high level of language aptitude and the experience of personal growth and self-fulfilment offered by the development of her linguistic skills, was enough to override the combination of the factors based on which the DA predicted low achievement in both languages for her.

As this and other cases in which the DA was unable to predict group membership show, the factors we included in our research design are not sufficient to paint a full picture of the circumstances under which both L1 maintenance and L2 acquisition may be more or less successful. Future studies should delve yet deeper into these questions and attempt to measure personal characteristics, such as language aptitude, and other aspects of attitude and motivation.

What the study presented above shows very clearly, however, is that investigations of language dominance cannot afford to adopt a one-dimensional perspective, nor to rely on linear models of predictor-outcome relationships. We hope that these findings may inform future studies and also encourage investigations that are able to zoom in on more specific linguistic features than the relatively global and holistic ones we were able to measure here, in order to further inform our understanding of bilingual development and the forces that drive and shape it.

## Author Contributions

MSS was responsible for data collection, classification and coding for Study 1, conducted all analyses reported here, and wrote the first draft of the article. GY was responsible for the data collection, classification and coding of the Turkish data in Study 2, and contributed to the revisions of the article.

## Conflict of Interest Statement

The authors declare that the research was conducted in the absence of any commercial or financial relationships that could be construed as a potential conflict of interest.

## Footnotes

1 The number of dimensions can be extended to encompass more than two languages. The discussion here will be confined to a two-language scenario for the sake of simplicity, but may equally be taken to refer to multilinguals proficient in a potentially unlimited number of languages.

2 The model further identifies subpopulations of ‘non-natives’: (a) early bilinguals and heritage speakers, (b) immersed second language learners and (c) instructed second language learners (Hulstijn, 2015: 47f.). For the purpose of the present study discussion will be restricted to Category B – those speakers who typically form the populations under investigation in attrition studies.

3 A full review of findings on L1At being beyond the scope of this paper we refer the reader to Schmid and Köpke (2017). We limit the features we discuss to relatively global measures that can be compared between a bilinguals’ two languages and exclude more language-specific features, such as tense and aspect (e.g., Montrul, 2008) or number agreement (Kasparian et al., 2017).

4 https://languageattrition.org/resources-for-researchers/experiment-materials/sociolinguistic-questionnaires/

5 All materials are available on https://languageattrition.org/resources-for-researchers/experiment-materials/
