# Supplementary material for: Predictors of Language Dominance: An Integrated Analysis of First Language Attrition and Second Language Acquisition in Late Bilinguals
Source: Front Psychol. 2018 Aug 20;9:1306. doi: 10.3389/fpsyg.2018.01306 (PMC6110303; doi:10.3389/fpsyg.2018.01306)
Supplement: Supplementary file 1 [file Table_1.pdf]

Table S1: Self-reported levels language proficiency, frequency of L1 use and language attitudes

		Germans in Canada (n = 53)	Germans in the Netherlands (n = 53)	Moroccans in the Netherlands (n = 35)	Turks in the Netherlands (n = 52)
Self-rated language proficiency					
L1 proficiency at time of testing	none/very bad				1
	bad	1			
	some	7	12	12	11
	good	17	29	6	32
	very good	28	12	17	8
L2 proficiency at time of testing	none/very bad				
	bad		3	1	8
	some	1	11	18	20
	good	14	20	15	18
	very good	38	19	1	6
L1 proficiency at time of emigration	none/very bad				
	bad				
	some			1	6
	good	7	18	6	35
	very good	46	35	28	11
L2 proficiency at time of emigration	none/very bad	9	49	35	52
	bad	10	1		
	some	21	2		
	good	6	1		
	very good	7			
Frequency of use of L1					
How frequently do you use your L1 overall?	rarely				1
	a few times a year	4	9		
	monthly	6	9		
	weekly	15	13	7	1
	daily	28	22	28	50
How frequently do you use your L1 at work?	never	34	29	27	35
	seldom	4	7	3	5
	sometimes	5	7	1	8
	often	6	8	1	3
	very often	4	2	3	1
How frequently do you use your L2 at work?	never		1	4	7
	seldom	1	2	1	7
	sometimes	2	5	1	9
	often	11	9	5	6
	very often	39	36	24	23
How frequently do you have contact with friends and family in your home country?	never	2	2		
	seldom	3	10		3
	sometimes	6	16	5	11
	often	19	17	7	28
	very often	23	8	23	10

How frequently do you visit your home country?	never	4			
	seldom	31			1
	sometimes	17			
	often		24		10
	very often	1	29	35	41
How frequently do you use your L1 with your partner?	average (0 = never, 1 = all the time)	.45	.43	.68	.89
How frequently do you use your L1 with your children?	average (0 = never, 1 = all the time)	.36	.43	.49	.75
How frequently do you use your L1 with your grandchildren?	average (0 = never, 1 = all the time)	.19	.16	.66	.10
How frequently do you use your L1 with your friends ad acquaintances?	average (0 = never, 1 = all the time)	.39	.34	.58	.66
What is the native language of most of your friends and acquaintances?	average (0 = all L2, 1 = all L1)	.28	.46	.62	.49
How frequently do you read books/newspapers, listen to the radio/music/watch TV in your L1?	average (0 = never, 1 = very often)	.45	.43	.68	.89
Attitudinal factors					
How important is it to you to maintain a good level of proficiency in your L1?	not important at all	3	1	1	
	not important	6	2		1
	neutral	2	5	1	2
	important	6	15	7	16
	very important	36	30	26	33
How important is it to you that your children should have a good level of knowledge of your L1?	not important at all	2	2	1	4
	not important	6	2	2	2
	neutral	5	3		4
	important	18	27	8	16
	very important	22	19	24	26
Which culture do you prefer?	only L2	2	1		
	more L2	15	14	1	6
	equal	16	17	21	15
	more L1	13	15	11	23
	only L1	7	6	2	8
Which language do you prefer?	L2	24	13	3	4
	equal	1	27	6	11
	L1	28	13	26	37
Do you consider yourself a balanced bilingual?	No, my L2 is better	15	10	1	2
	yes	30	28	4	12
	No, my L1 is better	8	15	30	38
