# Supplementary material for: Predictors of Language Dominance: An Integrated Analysis of First Language Attrition and Second Language Acquisition in Late Bilinguals
Source: Front Psychol. 2018 Aug 20;9:1306. doi: 10.3389/fpsyg.2018.01306 (PMC6110303; doi:10.3389/fpsyg.2018.01306)
Supplement: Supplementary file 2 [file Table_2.pdf]

Table S2: Principal Component Analysis – Rotated Component Matrix

	PCA Interactive use	PCA Personal background	PCA Perception	PCA Attitude	PCA Contact	PCA Professional use
Social interaction in L1	.784					
Frequency of L2 use with friends	-.711					
Frequency of L1 use with friends	.653					
L2 proficiency at time of testing	-.650					
Native language of most friends and acquaintances	.639					
Self-rated balanced bilingual	.632					
Language of preference	.597					
Frequency of L1 use within family	.553					
Overall frequency of L1 use	.500					
Age at testing		.851				
Length of Residence		.842				
Level of education		-.605				
Self-perceived change of L1			.743			
L1 proficiency at time of testing			.624			
Frequency of use of L1 media				.661		
Importance of maintaining L1				.623		
Importance that children should speak L1				.595	.533	
Culture of preference				.454		
Amount of contact with L1					.812	
Use of L1 for professional purposes						.866
Use of L2 for professional purposes						-.639

Extraction Method: Principal Component Analysis. Rotation Method: Varimax with Kaiser Normalization.
Rotation converged in 12 iterations.
