# Supplementary material for: Predictors of Language Dominance: An Integrated Analysis of First Language Attrition and Second Language Acquisition in Late Bilinguals
Source: Front Psychol. 2018 Aug 20;9:1306. doi: 10.3389/fpsyg.2018.01306 (PMC6110303; doi:10.3389/fpsyg.2018.01306)
Supplement: Supplementary file 3 [file Table_3.pdf]

Table S3: MANCOVA for HLC proficiency tasks in Study 1, separated by language

	GLM – L1				GLM – L2			
	Roy's largest root	F (2, 98)	p	Partial η^2	Roy's largest root	F (4, 89)	p	Partial η^2
Interactive Use					0.287	13.368	<.001	0.223
Personal Background					.105	4.865	<.05	0.095
Perception	0.184	9.034	<.001	0.156				
Attitude	0.215	10.534	<.001	0.177	0.068	3.161	<.05	.064
Professional Use	0.257	12.586	<.001	0.204	.134	6.221	<.01	.118
Contact								
