# Supplementary material for: Predictors of Language Dominance: An Integrated Analysis of First Language Attrition and Second Language Acquisition in Late Bilinguals
Source: Front Psychol. 2018 Aug 20;9:1306. doi: 10.3389/fpsyg.2018.01306 (PMC6110303; doi:10.3389/fpsyg.2018.01306)
Supplement: Supplementary file 4 [file Table_4.pdf]

Table S4: Discriminant Function Analysis, Study 1 – Structure Matrix

| | Function 1 | Function 2 | Function 3 |
|---|---|---|---|
| Frequency of use of L1 with the family | .605* | | |
| Overall frequency of use of L1 | .400* | | |
| Length of residence | -.345* | | |
| Frequency of L2 use with friends | -.343* | | |
| Frequency of L2 use at work | -.324* | | |
| Frequency of use of L1 media | .240* | | |
| Frequency of use of L1 with friends | .227* | | |
| Native language of friends and acquaintances | .221* | | |
| Frequency of contact with L1 speakers in home country | .196* | | |
| Importance that children should know L1 | .163* | | |
| Level of education | | .424* | |
| Frequency of L2 use at work | | .410* | |
| Self-reported proficiency in L2 at time of testing | | .374* | |
| Age at testing | | -.186* | |
| Self-perceived change to L1 | | | .400* |
| Culture of preference | | | .394* |
| Language of preference | | | .391* |
| Self-perceived bilingual balance | | | .373* |
| Language of friends | | | .332* |
| Self-reported proficiency in L1 at time of testing | | | .322* |
| Importance to maintain L1 | | | .142* |
