# Supplementary material for: Predictors of Language Dominance: An Integrated Analysis of First Language Attrition and Second Language Acquisition in Late Bilinguals
Source: Front Psychol. 2018 Aug 20;9:1306. doi: 10.3389/fpsyg.2018.01306 (PMC6110303; doi:10.3389/fpsyg.2018.01306)
Supplement: Supplementary file 5 [file Table_5.pdf]

Table S5: Discriminant Function Analysis, Study 2 – Structure Matrix

	Function 1	Function 2	Function 3
Language of preference	-.432*		
Frequency of L2 use with friends	.353*		
Self-reported proficiency in L2 at time of testing	.304*		
Frequency of use of L1 with the family	-.243*		
Native language of friends and acquaintances	-.216*		
Frequency of L1 use with friends	-.187*		
Culture of preference	-.169*		
Length of residence	.166*		
Self-perceived bilingual balance	-.128*		
Language of friends	-.110*		
Self-reported proficiency in L1 at time of testing		.326*	
Level of education		.284*	
Overall frequency of use of L1		.242*	
Frequency of use of L1 media		.227*	
Self-perceived change to L1		.120*	
Frequency of L1 use at work			.383*
Frequency of L2 use at work			.351*
Importance that children should know L1			-.271*
Frequency of contact with L1 speakers in home country			-.252*
Importance to maintain L1			-.226*
Age at testing			.117*
